# Mechanism of selective recruitment of RNA polymerases II and III to snRNA gene promoters

**DOI:** 10.1101/gad.314245.118

**Published:** 2018-05-01

**Authors:** Oleksandr Dergai, Pascal Cousin, Jerome Gouge, Karishma Satia, Viviane Praz, Tracy Kuhlman, Philippe Lhôte, Alessandro Vannini, Nouria Hernandez

**Affiliations:** 1 Center for Integrative Genomics, Faculty of Biology and Medicine, University of Lausanne, 1015 Lausanne, Switzerland;; 2 Division of Structural Biology, The Institute of Cancer Research, London SW7 3RP, United Kingdom;; 3 Swiss Institute of Bioinformatics, 1015 Lausanne, Switzerland;; 4 Cold Spring Harbor Laboratory, Cold Spring Harbor, New York 11724, USA

**Keywords:** BRF2, SNAPc, TBP, TFIIA, TFIIB, small nuclear RNA promoters

## Abstract

RNA polymerase II (Pol II) small nuclear RNA (snRNA) promoters and type 3 Pol III promoters have highly similar structures; both contain an interchangeable enhancer and “proximal sequence element” (PSE), which recruits the SNAP complex (SNAPc). The main distinguishing feature is the presence, in the type 3 promoters only, of a TATA box, which determines Pol III specificity. To understand the mechanism by which the absence or presence of a TATA box results in specific Pol recruitment, we examined how SNAPc and general transcription factors required for Pol II or Pol III transcription of SNAPc-dependent genes (i.e., TATA-box-binding protein [TBP], TFIIB, and TFIIA for Pol II transcription and TBP and BRF2 for Pol III transcription) assemble to ensure specific Pol recruitment. TFIIB and BRF2 could each, in a mutually exclusive fashion, be recruited to SNAPc. In contrast, TBP–TFIIB and TBP–BRF2 complexes were not recruited unless a TATA box was present, which allowed selective and efficient recruitment of the TBP–BRF2 complex. Thus, TBP both prevented BRF2 recruitment to Pol II promoters and enhanced BRF2 recruitment to Pol III promoters. On Pol II promoters, TBP recruitment was separate from TFIIB recruitment and enhanced by TFIIA. Our results provide a model for specific Pol recruitment at SNAPc-dependent promoters.

## Abstract (precis)

In this study, Dergai et al. sought to understand the mechanism by which the absence or presence of a TATA box results in specific polymerase recruitment. They examined how SNAPc and general transcription factors required for Pol II or Pol III transcription of SNAPc-dependent genes (i.e. TBP, TFIIB, and TFIIA for Pol II transcription and TBP and BRF2 for Pol III transcription) assemble to ensure specific polymerase recruitment and provide a model for specific Pol recruitment at SNAPc-dependent promoters.

Mammalian nuclear genomes are transcribed by three specialized RNA polymerases. RNA polymerase I (Pol I) generates the precursor of 28S, 18S, and 5.8S ribosomal RNA (rRNA); Pol II transcribes all protein-coding genes, long noncoding RNA genes, and most genes encoding small nuclear RNAs (snRNAs); and Pol III synthesizes short noncoding RNAs such as transfer RNAs (tRNAs), 5S rRNA, U6 snRNA, and other short untranslated RNAs. Each group of genes has particular promoter elements, gene body architecture, and termination signals.

Pol II promoters can be divided into mRNA TATA-containing, mRNA TATA-less, and snRNA (TATA-less) promoters. Assembly of a preinitiation complex (PIC) on mRNA TATA-containing promoters, which involves binding of TATA-box-binding protein (TBP) to the TATA box stabilized by TFIIA and TFIIB, has been extensively studied in vitro (Orphanides et al. 1996; Tan and Richmond 1998; Liu et al. 2013), and the structure of the yeast (Plaschka et al. 2016) and human (He et al. 2016) PIC leading to promoter opening has been determined recently by cryo-electron microscopy. In vivo, TBP is recruited to TATA-containing as well as TATA-less promoters as part of a large complex called TFIID, of which several subunits (in particular TBP-associated factor 1 [TAF1] and TAF2) contribute to binding to DNA (see Louder et al. 2016 and references therein). This role of TFIID subunits is particularly important for TBP positioning at TATA-less mRNA promoters. Pol II snRNA core promoters contain a proximal sequence element (PSE) (Hernandez 2001; Jawdekar and Henry 2008), which binds the five-subunit SNAP complex (SNAPc). In addition to SNAPc, basal transcription from Pol II snRNA promoters requires TBP, TFIIA, TFIIB, TFIIF, and TFIIE (Kuhlman et al. 1999) as well as some of the TFIID TAFs (Zaborowska et al. 2012). Pol II snRNA promoters also contain a distal sequence element (DSE), which enhances transcription from the core promoter (Hernandez 2001; Jawdekar and Henry 2008).

Pol III promoters can be divided into three broad categories. Types 1 and 2 are gene-internal and recruit the assembly factors TFIIIA and TFIIIC in the first case or TFIIIC directly in the second case. In turn, TFIIIC binds the Pol III-recruiting factor BRF1–TFIIIB, which is composed of three subunits: TBP, the TFIIB-related factor BRF1, and BDP1 (Geiduschek and Kassavetis 2001; Schramm and Hernandez 2002). Type 3 promoters, on the other hand, are gene-external and almost identical to Pol II snRNA promoters, comprising a DSE and a PSE, flanked in addition by a TATA box (Hernandez 2001). The core promoter recruits SNAPc (which binds to the PSE) and BRF2–TFIIIB (composed of TBP, which binds to the TATA box; the TFIIB-related factor BRF2, which replaces BRF1 in this complex; and BDP1). BRF1 and BRF2 are highly related in sequence to TFIIB in their N-terminal regions but in addition contain C-terminal extensions not present in TFIIB (Wang and Roeder 1995; Mital et al. 1996; Schramm et al. 2000). Crystal structures of BRF2–TFIIIB bound to a TATA box show that BRF2, like TFIIB, contacts TBP through a core domain composed of two cyclin repeats (Gouge et al. 2015) and, similarly to BRF1, (Juo et al. 2003), establishes additional contact through its C-terminal extension (Saxena et al. 2005; Gouge et al. 2015).

The Pol II snRNA promoters and the type 3 Pol III promoters thus have the particularity of sharing two of their promoter elements (the DSE and the PSE) as well as a number of transcription factors; i.e., DSE-binding factors, the PSE-binding factor SNAPc, and TBP. Although the PSEs of Pol II and Pol III transcribed genes differ slightly in their consensus sequences, they are interchangeable and do not contribute substantially to polymerase selection (Lobo and Hernandez 1989). On the contrary, addition of a TATA box downstream from the PSE in a Pol II snRNA promoter results in a shift to Pol III recruitment. Thus, in PSE-containing promoters, the presence of a TATA box determines polymerase recruitment specificity (Lobo and Hernandez 1989; James Faresse et al. 2012).

Although the predominant role of the TATA box in determining polymerase recruitment specificity at SNAPc-dependent promoters is clearly established, the mechanism by which specific polymerase recruitment is actually achieved is not understood. In particular, why is Pol II recruited to the TATA box of mRNA promoters but not the TATA box of Pol III SNAPc-dependent promoters? How are TBP and TFIIB recruited to SNAPc-dependent promoters in the absence of a TATA box? In general, how are RNA polymerase-specific PICs assembled at SNAPc-dependent promoters? To address these questions, we used minimal sets of transcription factors capable of nucleating specific PICs on Pol II or Pol III SNAPc-dependent promoters and examined the specificity of various individual protein–protein interactions. Our results reveal mutually exclusive protein–protein associations and a key role for TBP in both favoring BRF2 recruitment to TATA-containing promoters and preventing BRF2 recruitment to TATA-less promoters. Our results provide a model explaining how RNA polymerase specificity is achieved at snRNA promoters.

## Results

### Components of the Pol II and Pol III machineries present at snRNA gene promoters

**Figure 1.**
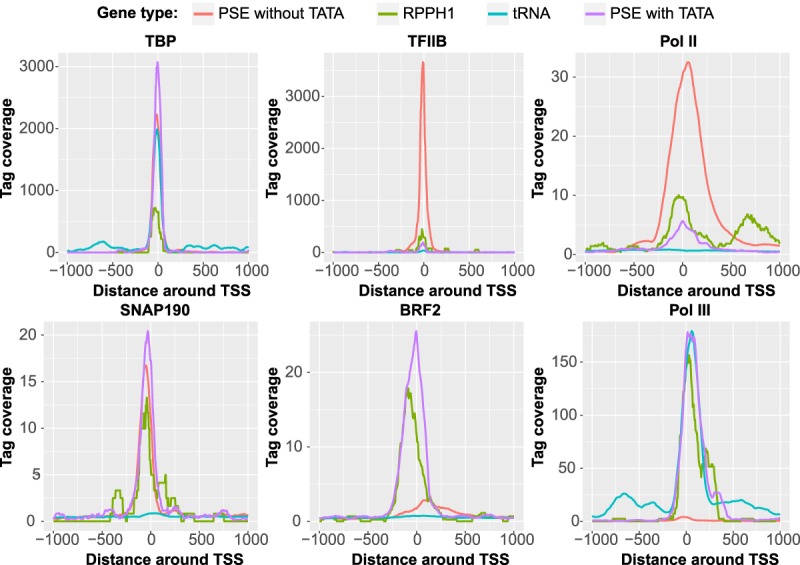
Average tag density plots for the indicated proteins around the TSSs of various gene families. Annotated PSE-containing genes were split into TATA-containing (purple) and TATA-less (red) genes, and only active genes, as determined by TBP occupancy, were kept for further analysis. The *RPPH1* gene (olive green) was analyzed separately. Only active annotated tRNA genes (turquoise), as determined by Pol III occupancy, were analyzed. See the Materials and Methods for further details.

We exploited publicly available ChIP-seq (chromatin immunoprecipitation [ChIP] combined with high-throughput sequencing) data to examine the distribution of Pol II and Pol III transcription factors at SNAPc-dependent promoters as well as at tRNA promoters. We selected active SNAPc-dependent promoters based on TBP occupancy and then separated them into TATA-containing (Pol III) and TATA-less (Pol II). The *RPPH1* gene, which can be occupied by either Pol II or Pol III (James Faresse et al. 2012), was analyzed separately. We plotted tag density profiles to determine the position of each protein relative to the transcription start site (TSS) as well as its relative abundance at the different types of promoters (Fig. 1; Supplemental Fig. 1). We found peaks of TBP at a very similar position close to the TSSs of all promoters analyzed, and the average tag densities were quite similar in Pol II and Pol III genes. Thus, regardless of the composition of assembled PICs, TBP is present, and its position relative to the TSS is conserved.

The TFIIB tag density profile resembled that of TBP but with a much higher tag density at Pol II genes as compared with Pol III genes. Nevertheless, we observed some TFIIB at TATA-containing Pol III SNAPc-dependent genes, whereas there was no detectable TFIIB at Pol III SNAPc-independent tRNA genes. Consistent with the TFIIB tag profiles, the Pol II tag profile was highest at Pol II snRNA genes, much lower but detectable at Pol III SNAPc-dependent genes, and undetectable at tRNA genes (Fig. 1). This finding prompted us to examine the distribution of C-terminal domain (CTD) phosphorylated species of Pol II (which reflect different states of the elongating enzyme) as well as that of DSIF, a factor involved in regulating Pol II elongation. For all of these features, we observed occupancy peaks right downstream from the TSS of Pol II SNAPc-dependent genes but not at Pol III SNAPc-dependent genes or, as expected, at tRNA genes (Supplemental Fig. 1). Thus, Pol II detected at Pol III TATA-containing SNAPc-dependent promoters probably corresponds to transiently misrecruited enzyme molecules rather than transcriptionally engaged ones.

TFIIA, TAF1, and TAF7 were all present at Pol II SNAPc-dependent promoters but not at Pol III promoters, with the TAF1 and TAF7 peaks located downstream from the TSS, as observed in mRNA promoters (Supplemental Fig. 1; Louder et al. 2016). This confirms the involvement of TFIIA in Pol II snRNA gene transcription (Kuhlman et al. 1999) and suggests that more of the TFIID subunits than previously reported (Zaborowska et al. 2012) are actually present at Pol II snRNA PICs. BRF2, BDP1, and the RPC4 Pol III subunit were specifically enriched at Pol III SNAPc-dependent genes and, in the two latter cases, at tRNA genes. BRF2 was not recruited to tRNA genes despite its high structural similarity to BRF1, consistent with previous results (Fig. 1; Supplemental Fig. 1; James Faresse et al. 2012). The accumulation profiles of two SNAPc subunits revealed a slightly decreased SNAP19 tag density and, as observed before (James Faresse et al. 2012), a downstream-shifted SNAP190 peak position at Pol III versus Pol II snRNA promoters, suggesting that the conformation of SNAPc might differ at Pol II and Pol III PSE-containing promoters (Fig. 1; Supplemental Fig. 1).

The tag densities observed at the *RPPH1* gene (green line in Fig. 1; Supplemental Fig. 1) are consistent with the gene being occupied by Pol II and Pol III, with a prevalence of Pol III, as described before (James Faresse et al. 2012). Importantly, although we did not detect TFIIA, perhaps due to the low amount of reads in the data set, we detected Pol II phosphorylated at Ser2, Ser5, and Ser7 within its CTD as well as TAF1 and TAF7, indicating that, unlike Pol II present at other SNAPc-dependent TATA-containing promoters, Pol II at the *RPPH1* gene is transcriptionally active. Thus, the *RPPH1* gene can be transcribed by either Pol III or, in fewer occurrences, Pol II in vivo.

### The structurally related TFIIB and BRF2 proteins can bind directly to SNAPc

**Figure 2.**
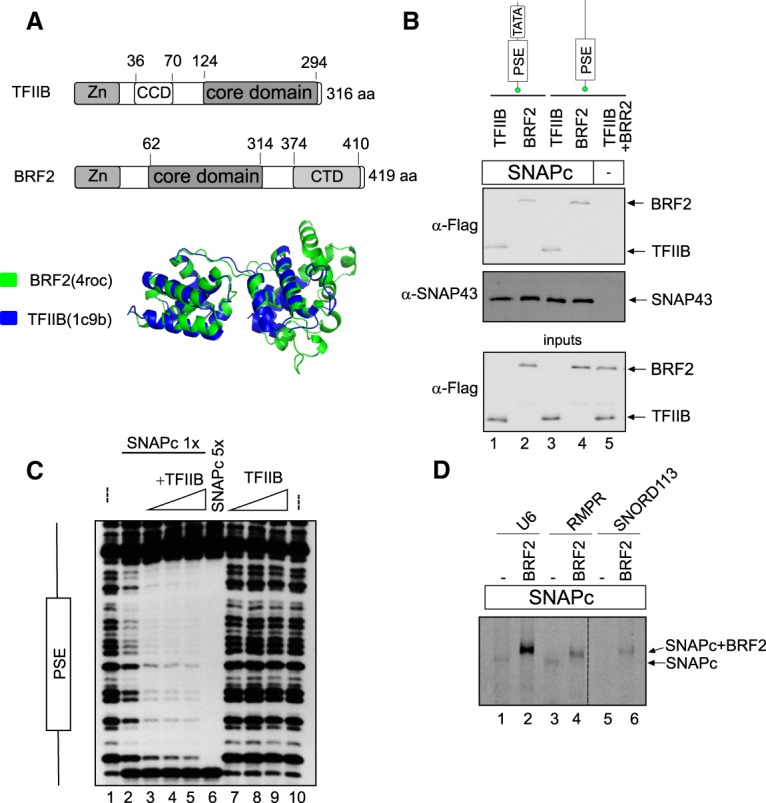
The structurally related TFIIB and BRF2 proteins can bind directly to SNAPc. (*A*, *top* panel) Modular structure of TFIIB and BRF2. (Zn) Zn ribbon domain involved in interaction with RNA polymerase. The core domain consists of two cyclin-like globular domains. (*Bottom* panel) Structural alignment of the BRF2 (green) and TFIIB (blue) core domains. 4roc and 1c9b refer to the Protein Data Bank (PDB) IDs (http://www.rcsb.org). (*B*) In vitro binding assay showing Flag-tagged BRF2 and TFIIB as detected by anti-Flag antibody (*top* panel) and SNAPc as detected by an anti-SNAP43 antibody (*middle* panel) retained on biotinylated DNA probes containing the mouse *U6* PSE and TATA box or just the mouse *U6* PSE bound to streptavidin beads. The *bottom* panel shows input proteins. (*C*) DNase I footprinting assay performed on a probe containing the mouse *U6* PSE and either no added proteins (lanes *1*,*10*), a low amount of SNAPc (lanes *2*–*5*) with no (lane *2*) or increasing amounts (lanes *3*–*5*) of TFIIB, a five times higher amount of SNAPc (lane *6*), or no SNAPc and increasing amounts of TFIIB (lanes *7*–*9*). (*D*) SNAPc alone (lanes *1*,*3*,*5*) or combined with BRF2 (lanes *2*,*4*,*6*) was mixed with fluorescently labeled DNA probes containing the PSE (and, for *U6*, the TATA box) of the *U6*, *RMRP*, and *SNORD113* promoters, as indicated *above* the lanes, and analyzed by electrophoretic mobility shift assay (EMSA). The SNAPc–PSE and SNAPc–BRF2–PSE complexes are indicated. The SNAPc–PSE complex is visible in lanes *1* and *3*.

On snRNA promoters, TFIIB and BRF2 are required to recruit Pol II and III, respectively (Kuhlman et al. 1999; Schramm and Hernandez 2002; James Faresse et al. 2012). TFIIB and BRF2 belong to the same protein family and share structural homology. Both proteins contain an N-terminally located Zn ribbon domain that constitutes an RNA polymerase interaction module (Buratowski and Zhou 1993; Saxena et al. 2005) and a similar core domain (Fig. 2A; Gouge et al. 2015). TFIIB contains a specific charged cluster domain (CCD) that interacts with the core domain and modulates its ability to assemble into a PIC (Hayashi et al. 1998; Glossop et al. 2004). BRF2 contains a specific CTD (Saxena et al. 2005) that is absent in TFIIB. These proteins were not retained on beads coated with a PSE-containing probe on their own but did bind when SNAPc was present (Fig. 2B, lanes 3–5, note that the presence of SNAPc is inferred from detection of its SNAP43 subunit). The presence of a TATA box in addition to the PSE did not affect these interactions (Fig. 2B, lanes 1,2). The amounts of TFIIB and BRF2 retained on SNAPc were similar, suggesting similar affinities. Thus, both TFIIB and BRF2 can bind to a SNAPc–PSE complex.

Addition of TFIIB enhanced in a dose-dependent manner the footprinting of SNAPc to a PSE as measured by DNase I protection assay (Fig. 2C, lanes 2–5). Addition of BRF2 in an electrophoretic mobility shift assay (EMSA) supershifted and increased the amount of the SNAPc–PSE complex (Fig. 2D) on PSEs from Pol III (*U6* and *RMPR*) and Pol II (*SNORD113*) promoters. Thus, both TFIIB and BRF2 enhance loading of SNAPc to a PSE, and, at least for BRF2, the effect is independent of the Pol II or III promoter origin of the PSE.

### TBP directs specific recruitment of Pol II and III at snRNA promoters

The efficient recruitment of TFIIB as well as that of BRF2 to a SNAPc–PSE complex indicates that these factors alone are not sufficient to trigger specific assembly of a Pol II or Pol III transcription initiation complex. On the other hand, the presence of a TATA box downstream from the PSE strongly favors the recruitment of Pol III over Pol II, whereas its absence favors the recruitment of Pol II (Lobo and Hernandez 1989). This is not due to the presence or absence of TBP in the PIC, since TBP is present close to the TSS (Fig. 1)—and is required for transcription—of both Pol II and Pol III PSE-containing promoters (Kuhlman et al. 1999). Thus, it is apparently the recognition of the TATA box by TBP rather than TBP recruitment per se that plays a role in directing the specific assembly of a Pol III versus a Pol II PIC. To test this idea, we prepared binding reactions containing (1) biotinylated mouse *U6* snRNA promoter fragments with combinations of intact or mutated PSE and TATA box, (2) SNAPc as a nucleating transcription factor, and (3) both Flag-tagged TFIIB and BRF2, recruiters of Pol II and Pol III, respectively, and, in some cases, TBP. We then collected the DNA fragments on streptavidin beads and detected bound proteins by immunoblot.

**Figure 3.**
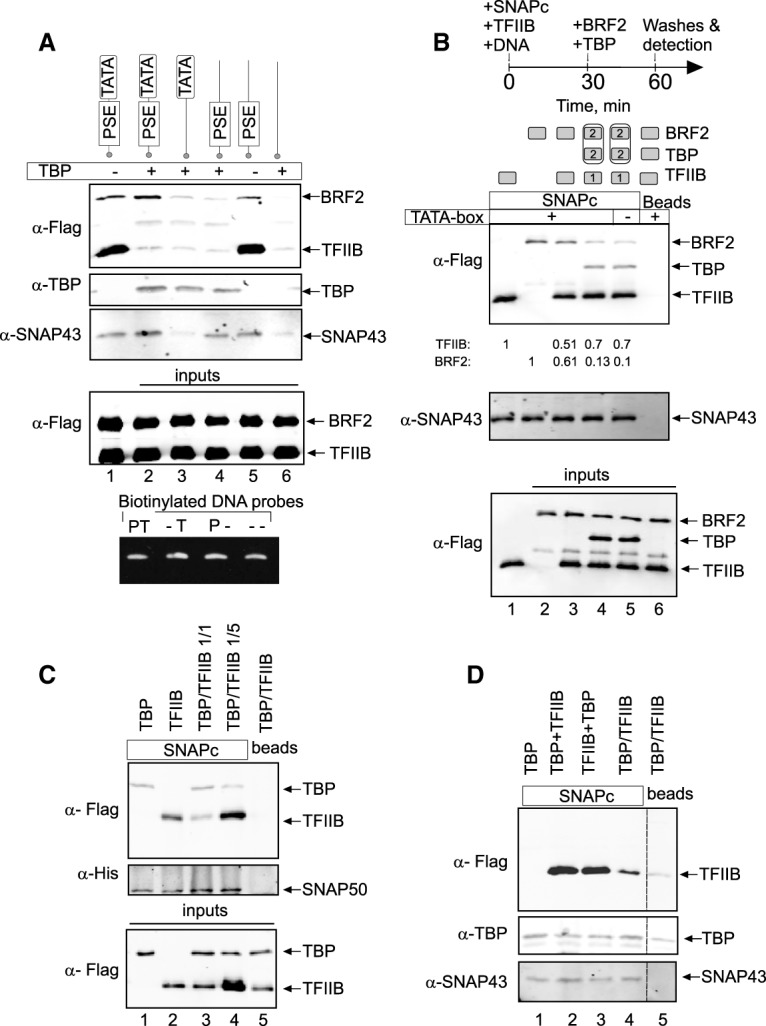
TBP directs specific recruitment of Pol II and III at SNAPc-dependent promoters. (*A*) SNAPc and both Flag-tagged TFIIB and BRF2 were mixed with, in some cases, TBP and biotinylated DNA probes containing various combinations of PSE and TATA box from the mouse *U6* snRNA promoter, as indicated at the *top*. (*Top* three panels) DNA fragments were collected on streptavidin beads, and bound proteins were detected by immunoblot with the indicated antibodies. The two *bottom* panels show input proteins and DNA probes. (PT) PSE and TATA box are intact; (–T) PSE is mutated, and TATA box is intact; (P–) PSE is intact, and TATA box is mutated; (– –) both PSE and TATA box are mutated. (*B*) BRF2 and TFIIB compete for binding to SNAPc. The proteins indicated at the *top* were mixed with biotinylated DNA probe containing a PSE and, where indicated *above* the lanes, a TATA box, and the experiment was performed as in *A*. The cartoon at the *top* of the panel refers to lanes *4* and *5*, where BRF2 and TBP on the one hand and TFIIB, SNAPc, and the DNA probe on the other hand were incubated separately for 30 min before being mixed together and incubated for another 30 min. The two *top* panels indicate proteins bound to the DNA probes, and the *bottom* panel shows input proteins. The numbers *below* the *top* panel indicate the relative amounts of TFIIB and BRF2 with respect to the first two lanes. (*C*) Binding of TBP and TFIIB to SNAPc immobilized on Strep-Tactin beads. The proteins added to the beads are shown at the *top*. In lanes *3* and *4*, TFIIB and TBP in ratios of 1:1 or 1:5, as indicated, were added simultaneously to the beads. The two *top* panels show bound proteins, and the *bottom* panel shows input proteins. (*D*) As in *C*, but TBP and TFIIB were added either simultaneously (lanes *4*,*5*) or sequentially, with TBP added before TFIIB (lane *2*) or TFIIB added before TBP (lane *3*).

SNAPc was efficiently recruited by PSE-containing DNA probes, but not by probes lacking the PSE, regardless of the presence of TBP or a TATA box (Fig. 3A, panels 2,3). Thus, the PSE is the main element recruiting SNAPc; in its absence, even a TBP–TATA-box complex cannot efficiently recruit SNAPc (Fig. 3A, lane 3). TBP, on the other hand, was recruited to not only probes containing a TATA box but also (in these binding reactions containing SNAPc, BRF2, and TFIIB) a probe containing only a PSE (Fig. 3A, panel 2, lanes 2–4). This suggests a direct SNAPc–TBP interaction, which might contribute to TBP recruitment to TATA-less Pol II snRNA promoters.

TFIIB was efficiently recruited to DNA probes containing a PSE with or without a TATA box, but, strikingly, this recruitment was strongly decreased in the presence of TBP (Fig. 3A, panel 1, cf. lanes 1 and 2 and lanes 5 and 4). As shown below, this TBP-directed inhibition of TFIIB binding to a SNAPc–PSE complex most likely results from TBP and TFIIB forming a complex off the DNA, which is incompetent for binding to SNAPc. For BRF2, maximum recruitment was on a probe containing a PSE and a TATA box in the presence of both SNAPc and TBP (Fig. 3A, panel 1, lane 2), consistent with cooperative binding of SNAPc and TBP to their respective DNA-binding sites (Mittal and Hernandez 1997; Ma and Hernandez 2002) and with the cooperative BRF2–SNAPc binding shown above (Fig. 2D). Indeed, BRF2 binding to just the TBP–TATA-box complex was much weaker (Fig. 3A, cf. lanes 2 and 3). BRF2 could also bind to just the SNAPc–PSE complex, although less strongly than TFIIB, but, importantly, this binding was attenuated by the presence of TBP (Fig. 3A, panel 1, cf. lanes 5 and 4). Thus, TBP has a bimodal impact on BRF2 recruitment to snRNA promoters: It enhances (slightly) BRF2 association with a SNAPc–PSE complex on TATA-containing probes (Fig. 3A, lanes 1,2) and inhibits it on TATA-less ones (Fig. 3A, lanes 4,5). This latter effect may reflect the same phenomenon as observed for TFIIB; i.e., formation of a BRF2–TBP complex off the DNA, which is then unable to bind to the SNAPc–PSE complex on probes lacking a TATA box.

We further explored TFIIB versus BRF2 recruitment to a SNAPc–PSE complex. When TFIIB and BRF2 were added simultaneously to a SNAPc–PSE complex, the amount of each bound protein was slightly less than when added separately, suggesting that TFIIB and BRF2 compete for the same SNAPc interface (Fig. 3B, top panel, lanes 1–3). We then tested adding a preformed BRF2–TBP complex to a preformed TFIIB–SNAPc–PSE complex (see the cartoon at the top of Fig. 3B). In this case, most of the TFIIB remained bound to SNAPc with very little recruitment of BRF2 (13% and 10%, respectively) (Fig. 3B, top panel, cf. lanes 4,5 and the reaction with no competition in lane 2) whether or not a TATA box was present in the probe. These results suggest that (1) PSE-bound SNAPc can accommodate only one molecule, either TFIIB or BRF2, and (2) the TFIIB–SNAPc–PSE complex is quite stable such that once it is formed, efficient recruitment of a TBP–BRF2 complex is prevented even in the presence of a TATA box.

### The SNAPc- and TBP-interacting surfaces of TFIIB overlap

We observed above that TBP prevented efficient binding of TFIIB to a SNAPc–PSE complex (Fig. 3A). We further examined the abilities of TBP and TFIIB to interact with SNAPc in the absence of DNA in an in vitro binding assay in which SNAPc is directly attached to beads. TFIIB and TBP each bound SNAPc in this assay when added separately (Fig. 3C, lanes 1,2) or consecutively (Fig. 3D, lanes 2,3). However, when they were first mixed together and then added to the SNAPc beads, we observed strongly decreased TFIIB binding to SNAPc (Fig. 3C [cf. lanes 2 and 3], D, cf. lanes 2,3 and 4), which could be restored by adding a five times molar excess of TFIIB (Fig. 3C, lanes 2–4). These results are consistent with the results above (Fig. 3A) and with the idea that once bound to TBP in a binary complex, TFIIB cannot bind to SNAPc.

**Figure 4.**
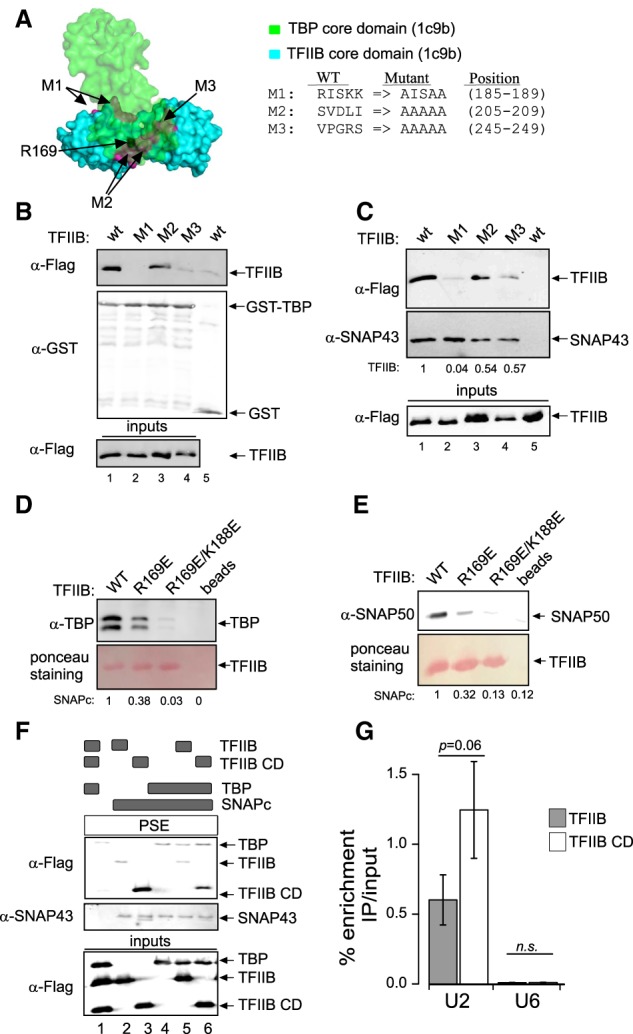
The SNAPc- and TBP-interacting surfaces of TFIIB overlap, and the TFIIB N-terminal domain negatively impacts TFIIB recruitment to snRNA gene promoters. (*A*) The TBP and TFIIB core domain complex (PDB ID: 1c9b), with the three patches of TFIIB residues mutated to alanines indicated in magenta, residue R169 shown in black, and the alanine substitutions shown at the *right*. (*B*) TFIIB wild type or mutant, as indicated *above* the lanes, was mixed with GST-TBP (lanes *1*–*4*) or just GST (lane *5*) attached to beads, and bound proteins were detected by immunoblotting with the antibodies indicated at the *left*. The *bottom* panel shows the wild-type and mutant TFIIB inputs. (*C*) As in *B*, but SNAPc rather than TBP was immobilized on beads. (*D*,*E*) TBP (*D*) or SNAPc (*E*) was incubated with Flag-tagged TFIIB or its mutants immobilized on Flag beads, and bound proteins were detected by immunoblotting with the indicated antibodies. (*F*) The proteins, as indicated *above* the lanes by black rectangles, were mixed with a PSE-containing probe attached to streptavidin beads. Bound proteins were detected by immunoblotting with the antibodies indicated at the *left*. The *bottom* panel shows input proteins. (*G*) ChIP-qPCRs (ChIP combined with quantitative PCRs) performed with anti-Flag antibodies in doxycycline-treated cell lines expressing doxycycline-inducible wild-type TFIIB or the TFIIB core domain. Enrichment immunoprecipitation/input at the *U2* and *U6* promoters is shown as mean ± SD of three independent experiments.

The idea that a TFIIB–TBP complex cannot bind to SNAPc implies mutually exclusive contacts between TFIIB and TBP and between TFIIB and SNAPc. We used the known structure of the TBP–TFIIB complex bound to a TATA box as found at mRNA promoters (Protein Data Bank [PDB] ID 1C9B) (Tsai and Sigler 2000) to design triple or quintuple alanine substitutions in the TFIIB core domain that were expected to affect binding to TBP. We introduced mutations in three different patches in the N-terminal or C-terminal cyclin-like repeat (M1 and M3) located in proximity to TBP and in the linker between the two repeats (M2) (Fig. 4A). These mutations indeed disrupted association with TBP in a GST-TBP pull-down assay, with the M1 and M3 mutations having a much larger effect than M2 (Fig. 4B). Strikingly, the M1 and M3 mutations also strongly reduced binding to SNAPc immobilized on beads, whereas the M2 mutation had only a slight negative effect (Fig. 4C). We designed two additional mutations: a single R169E substitution, which has been shown to prevent association with a TBP–TATA-box complex (Tansey and Herr 1997), and a double R169E/K188E substitution. Both mutations reduced TFIIB binding to TBP, with the double substitution having a stronger effect (Fig. 4D), and, again, the same pattern was observed for binding to SNAPc (Fig. 4E). The similar binding patterns of the TFIIB mutants toward TBP and SNAPc strongly suggest that the same TFIIB surface, which includes residues from the two cyclin-like repeats, is involved in binding to TBP in a binary TBP–TFIIB complex and in binding to SNAPc, consistent with the idea that once engaged in a complex with TBP, TFIIB cannot interact with SNAPc. This in turn suggests that the two proteins are recruited sequentially to SNAPc to form a trimeric complex.

### The TFIIB N-terminal domain negatively impacts TFIIB recruitment to snRNA gene promoters

The CCD region of the TFIIB N-terminal domain (see Fig. 2A) interacts with the TFIIB core domain, maintaining the protein in a “closed” conformation (Hayashi et al. 1998; Glossop et al. 2004). A truncated TFIIB containing just the core domain bound more efficiently to a SNAPc–PSE complex than the wild-type protein (Fig. 4F, lanes 2,3), consistent with TFIIB lacking the CCD and thus in an “open” conformation being more efficiently recruited to a SNAPc–PSE complex. In contrast, BRF2 lacking the N-terminal domain bound with an efficiency very similar to that of full-length BRF2 (Supplemental Fig. 2). Upon addition of TBP, the binding of both TFIIB and the TFIIB core domain to a SNAPc–PSE complex was reduced (Fig. 4F, lanes 5,6), consistent with this inhibition reflecting formation of a TFIIB–TBP complex, which does not require the TFIIB N-terminal domain (Nikolov et al. 1995). In line with the finding that the TFIIB core domain binds more strongly to SNAPc than full-length TFIIB, TFIIB core domain occupancy was elevated compared with that of TFIIB at the Pol II *U2* snRNA gene, but not at the Pol III *U6* snRNA gene, in cell lines overexpressing similar amounts of the TFIIB core domain or TFIIB (Fig. 4G; Supplemental Fig. 2B).

### The same TBP surface contacts SNAPc and TFIIB

**Figure 5.**
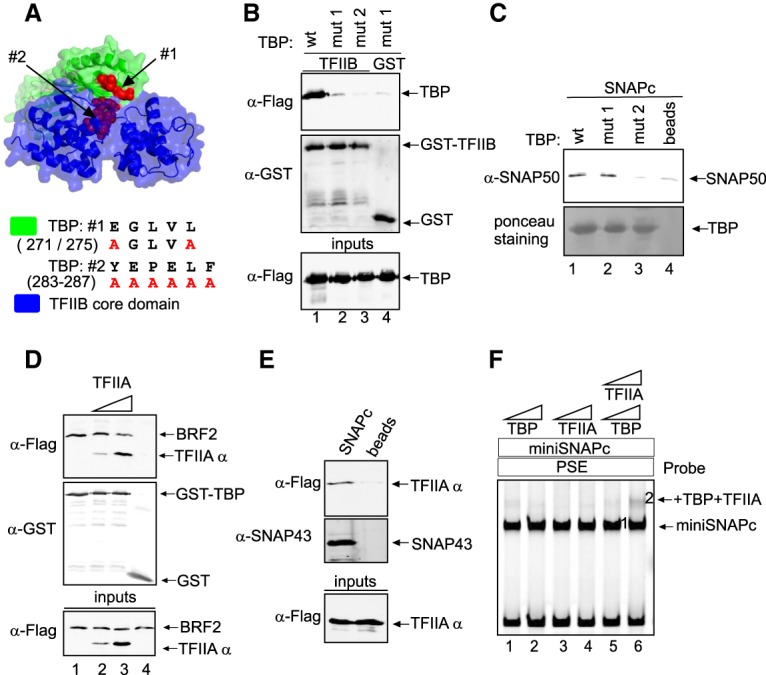
The SNAPc- and TFIIB-interacting surfaces of TBP partially overlap, and TFIIA prevents association of BRF2 with SNAPc and binds to SNAPc cooperatively with TBP. (*A*) Complex of the TBP and TFIIB core domains (PDB ID: 1c9b), with the two patches of TBP residues mutated to alanines indicated in red, and the alanine substitutions shown at the *right*. (*B*) Wild-type or mutant TBPs, as indicated *above* the lanes, were mixed with GST-TFIIB (lanes *1*–*3*) or just GST (lane *4*) attached to beads, and bound proteins were detected by immunoblotting with the antibodies indicated at the *left*. The *bottom* panel shows wild-type and mutant TBP inputs. (*C*) As in *B*, but SNAPc was mixed with TBP wild type or mutant immobilized on beads or just beads, as indicated *above* the lanes, and the *bottom* panel shows wild-type or mutant TBP bound to beads as detected by Ponceau staining. (*D*) As in *B*, but BRF2 and TFIIA, as indicated *above* the lanes, were mixed with GST-TBP (lanes *1*–*3*) or just GST (lane *4*). The *bottom* panel shows BRF2 and TFIIA (as detected by its TFIIAα subunit) inputs. (*E*) A PSE-containing biotinylated DNA probe was combined with SNAPc and TFIIA (lane *1*) or just TFIIA (lane *2*). DNA-associated protein complexes were collected on streptavidin beads, and bound proteins were detected by immunoblots with the antibodies indicated at the *left*. The *bottom* panel shows input protein. (*F*) EMSA performed with a mouse *U6* PSE DNA probe and the proteins indicated at the *top*. TBP and TFIIA were added in two concentrations: 1× and 3×.

TBP is required for Pol II transcription from SNAPc-dependent promoters, but our results indicate that it is not recruited as a preformed TBP–TFIIB complex. In Pol III snRNA promoters, TBP and SNAPc bind cooperatively to their respective DNA-binding sites (namely, the TATA box and the PSE), and this cooperative binding requires the N-terminal domain of TBP (Mittal and Hernandez 1997). However, in a GST pull-down assay, we found that the TBP core domain was as efficient as full-length TBP in binding SNAPc and that both proteins were much more efficient than truncations consisting of just part of the TBP N-terminal domain (Supplemental Fig. 3A,B). This indicates that the core domain of TBP can associate directly with SNAPc. We mutated residues to alanines in two separate regions of the TBP core domain—an α-helix surface (region 1) and a conserved loop (region 2) that are directly involved in interactions with TFIIB—and tested the resulting proteins for binding to GST-TFIIB or SNAPc immobilized on beads (Fig. 5A). The mutations in both patches strongly diminished TBP interaction with TFIIB, as expected (Fig. 5B). In addition, mutations in TBP region 2 strongly diminished TBP binding to SNAPc (Fig. 5C). Thus, mutation of region 2, directly involved in contacts with TFIIB in the TBP–TFIIB complex, also affects binding to SNAPc, indicating that this very TBP surface contacts SNAPc in a TBP–SNAPc complex. This is again consistent with the inability of a preformed TBP–TFIIB to bind to SNAPc and thus with formation of the SNAPc–TBP–TFIIB complex involving separate or sequential recruitment of TBP and TFIIB.

### TFIIA prevents association of BRF2 with SNAPc and binds to SNAPc cooperatively with TBP

TFIIA is required for Pol II snRNA gene transcription in vitro (Kuhlman et al. 1999), and structural data have shown that the TFIIA-interacting region of TBP coincides with the TBP region contacted by the BRF2 C-terminal extension (Gouge et al. 2015). In a GST-TBP pull-down assay, all three TFIIA subunits were retained on GST-TBP beads, as expected (Supplemental Fig. 3C), and increased TFIIA binding and concomitant decreased BRF2 binding were observed upon addition of increasing amounts of TFIIA, consistent with BRF2 and TFIIA binding to TBP being mutually exclusive (Fig. 5D). Additionally, TFIIA bound to SNAPc immobilized on beads (Fig. 5E) and led to a 60% increase in SNAPc recruitment to GST-TBP when added to the reaction mix (Supplemental Fig. 3D). A similar cooperative binding could be observed in an EMSA where, on a probe with a PSE (and lacking a TATA box), addition of either TBP or TFIIA alone to a SNAPc–PSE complex did not cause a visible supershift, whereas addition of both proteins together did (Fig. 5F). Together, these data suggest that TFIIA both prevents TBP association with BRF2 and facilitates TBP recruitment to SNAPc on TATA-less PSE-dependent promoters.

### TBP ensures proper BRF2 recruitment

We observed above that, similar to TFIIB, TBP inhibited BRF2 recruitment to a SNAPc–PSE complex on a probe lacking a TATA box but, unlike TFIIB, enhanced BRF2 recruitment to this complex on probes containing a TATA box (Fig. 3A). A key difference in the interaction of TFIIB and BRF2 with TBP bound to a TATA box is the primary TBP-binding site uniquely present in the C-terminal extension of BRF2 (Gouge et al. 2015). We generated a variant of BRF2 with residues D386 and E388 in the C-terminal extension mutated to alanines (DE → A) and therefore deficient in binding to TBP (Gouge et al. 2015, 2017). We then tested it for binding to a SNAPc–PSE complex on probes with or without a TATA box and in the presence or absence of TBP.

**Figure 6.**
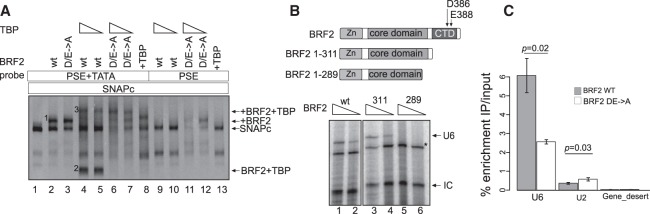
TBP ensures proper BRF2 recruitment. (*A*) EMSA performed with the DNA probes and protein factors SNAPc, wild-type or mutant BRF2 proteins, and TBP, as indicated at the *top* of the panel. (*B*, *top* panel) Structure of various BRF2 truncations. The position of amino acid A311 as well as those of D386 and E388, which are mutated in the DE → A mutant, are shown. (*Bottom* panel) In vitro transcription followed by T1 RNase protection assay was performed with decreasing amount of BRF2 wild type or truncated mutants. (U6) Protected fragment corresponding to correctly initiated U6 RNA; (IC) internal control; (*) nonspecific signal. (*C*) ChIP-qPCRs performed with anti-Flag antibodies in doxycycline-treated cell lines expressing doxycycline-inducible wild-type or mutant BRF2. Enrichment immunoprecipitation/input at the *U6* and *U2* genes. Data are shown as mean ± SD of three experiments.

Like wild-type BRF2, the DE → A mutant bound to a SNAPc–PSE complex to form a BRF2–SNAPc–PSE complex (Fig. 6A, lanes 1–3, complex 1). On probes containing a PSE and a TATA box, addition of TBP caused the appearance of a TBP–BRF2 complex as well as a TBP–BRF2–SNAPc complex (Fig. 6A, lanes 4,5, complexes 2 and 3). When BRF2 was replaced by the DE → A mutant, the TBP–BRF2 complex was disrupted, as expected, but the TBP–BRF2–SNAPc complex was still formed (Fig. 6A, cf. lanes 4,5 and 6,7, complexes 2 and 3). Thus, the BRF2–SNAPc interactions, which are not affected by the DE → A mutation (Fig. 6A, lanes 2,3, complex 1), can compensate for the loss of BRF2–TBP interactions in assembly of the TBP–BRF2–SNAPc complex. Indeed, a mutant BRF2 lacking most of the CTD (BRF2 1–311) could still direct Pol III transcription from a *U6* promoter in an in vitro transcription system. In contrast, a truncation lacking the entire CTD as well as the linker between the N-terminal domain and CTD did not (Fig. 6B). The linker has been shown to fold into an unusual semicircular α helix (the “arch”) that constitutes an interaction domain with SNAPc (Gouge et al. 2015). Thus, the results are consistent with the idea that the arch-mediated BRF2–SNAPc interaction can compensate, at least in vitro, for the loss of the BRF2–TBP interaction.

Importantly, on a probe with a wild-type PSE but lacking a TATA box, addition of TBP to a reaction containing BRF2 and SNAPc prevented assembly of a BRF2–SNAPc–PSE complex, whereas addition of TBP to a reaction containing the DE → A mutant BRF2 unable to bind to TBP still allowed some assembly of this complex (Fig. 6A, lanes 9–12, complex 1). This strongly argues that the inhibitory effect of TBP on recruitment of BRF2 to a SNAPc–PSE complex in the absence of a TATA box results from formation of a TBP–BRF2 complex, which is then unable to associate with the SNAPc–PSE complex in the absence of a TATA box.

To test the role of BRF2–TBP interactions in vivo, we generated doxycycline-inducible HEK293 cell lines expressing either wild-type or DE → A BRF2 (Supplemental Fig. 4) and performed ChIP-qPCR (ChIP combined with quantitative PCR) analysis with an anti-Flag antibody. We observed decreased occupancy of the DE → A mutant relative to wild-type BRF2 at the *U6* promoter and slightly increased occupancy at the *U2* gene (Fig. 6C). Thus, the BRF2–TBP interaction contributes in vivo, like in vitro, to selective BRF2 recruitment to Pol III SNAPc-dependent promoters.

## Discussion

SNAPc-dependent promoters comprise all of the Pol II snRNA promoters as well as all of the type 3 Pol III promoters, which include the U6 snRNA promoter. What specifies Pol II or Pol III recruitment in SNAPc-dependent promoters is the absence or presence, respectively, of a TATA box at a fixed distance downstream from the PSE. We used available ChIP-seq data to extend previous studies examining which basal transcription factors are present at Pol II and Pol III SNAPc-dependent promoters. On mRNA Pol II promoters, TBP is recruited as part of the TFIID complex, which contains a number of TAFs. We analyzed TAF1 and TAF7 occupancy data sets, as Zaborowska et al. (2012) reported the presence of TAF5, TAF6, TAF8, TAF9, TAF11, and TAF13—but not TAF1, TAF2, TAF3, TAF4, TAF7, TAF10, and TAF12—at Pol II snRNA promoters. Our analysis indicates the presence of these two TAFs at Pol II snRNA promoters, suggesting that the TFIID used at Pol II snRNA and mRNA mammalian promoters may not be different. Perhaps the different ChIP results reflect different TFIID conformations, which may have different cross-linking efficiencies. We were also interested in TFIIA because a previous study had suggested that this factor is required for transcription from Pol II SNAPc-dependent promoters (Kuhlman et al. 1999). Indeed, TFIIA was clearly detectable at Pol II SNAPc-dependent promoters, consistent with the idea that it is part of Pol II PICs assembled on PSEs.

Our analysis of ChIP-seq data indicates that SNAPc-dependent promoters are amazingly specific in their recruitment of polymerase in vivo. Thus, Pol III, BDP1, and BRF2 were not detectable at Pol II SNAPc-dependent promoters. Very small amounts of TFIIB and Pol II could be detected at Pol III SNAPc-dependent promoters, but DSIF as well as Pol II CTD phosphorylated residues were all undetectable, suggesting that any Pol II misrecruited to Pol III SNAPc-dependent promoters remains transcriptionally inactive. The *RPPH1* promoter is an interesting exception. It was reported before as recruiting both TFIIB and Pol II and BRF2 and Pol III (James Faresse et al. 2012). We confirmed these results and further showed the presence of TAF1, TAF7, and DSIF as well as Pol II phosphorylated on its CTD. These results strongly suggest that the *RPPH1* gene is unique among SNAPc-dependent genes in being able to direct the assembly of either an active Pol II or an active Pol III PIC.

### Specific assembly of Pol II and Pol III PICs on PSE-containing promoters

**Figure 7.**
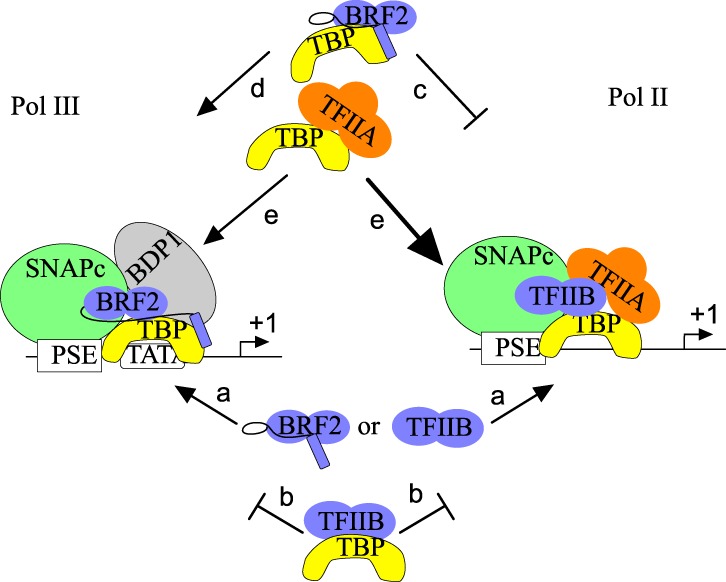
Model summarizing the recruitment of BRF2 and TFIIB to SNAPc-dependent promoters with or without a TATA box. (Arrows labeled a) BRF2 or TFIIB can join a SNAPc–PSE complex on probes with or without a TATA box. (Arrows labeled b) A TFIIB–TBP complex cannot join a SNAPc–PSE complex on probes with or without a TATA box. (Arrows labeled c and d) A BRF2–TBP complex cannot join a SNAPc–PSE complex on a probe without a TATA box (arrows labeled c) but can do so on a probe with a TATA box (arrows labeled d). (Arrows labeled e) A TBP–TFIIA complex can join a SNAPc–PSE complex on probes with and without a TATA box; however, it competes with BRF2, and thus recruitment when BRF2 is present is inefficient (and results in displacement of BRF2), whereas it helps recruitment of TBP to a SNAPc–PSE complex on probes without a TATA box and there prevents erroneous recruitment of BRF2. See the text for description.

To decipher the mechanisms underlying the remarkable specificity of SNAPc-dependent promoters, we examined protein–protein interactions that lead to assembly of Pol II and Pol III SNAPc-containing PICs. We found that in the absence of TBP, both TFIIB and BRF2 can be recruited to a SNAPc–PSE complex (Fig. 7, arrows labeled a); indeed, SNAPc and TFIIB or SNAPc and BRF2 bind cooperatively to a PSE. An interesting difference between TFIIB and BRF2 is the much more efficient binding displayed by TFIIB, but not BRF2, lacking the N-terminal domain as compared with the full-length protein. This may be related to the presence of the CCD in the TFIIB N-terminal domain (Glossop et al. 2004), which has been reported to mediate an intramolecular contact with the core domain. For mRNA promoters, this contact is thought to be released by activation factors such as Sp1 or VP16 interacting with the core domain and thus triggering a conformational change to an open conformation (Hawkes et al. 2000; Zheng et al. 2004). Our results suggest that for Pol II snRNA promoters, a similar mechanism may help efficient recruitment of TFIIB to SNAPc, since the DSE (located upstream of the PSE) recruits various transcription activators that may trigger a TFIIB open conformation. It remains that, on their own, the SNAPc–TFIIB and SNAPc–BRF2 interactions do not constitute a mechanism to favor a path to Pol II and Pol III recruitment, although, because the binding of TFIIB and BRF2 to SNAPc is mutually exclusive, they do ensure that a single type of polymerase (Pol II or Pol III) is recruited. Specific Pol II or Pol III PIC assembly is afforded by two additional factors: TBP (in conjunction with presence or absence of a TATA box) and TFIIA.

### Role of TBP and the TATA box in specific Pol II and III PIC assembly

Addition of TBP to binding reactions has two remarkable effects. First, the recruitment of TFIIB to SNAPc is inhibited whether or not a TATA box is present on the probe (Fig. 7, arrows labeled b). Our observations (1) that the TFIIB surfaces required for binding to SNAPc and TBP overlap (Fig. 4A–C) and (2) that the TBP surfaces required for binding to SNAPc and TFIIB partially overlap (Fig. 5A–C) suggest that addition of TBP results in the formation of a TFIIB–TBP dimeric complex, which is unable to bind to SNAPc. This puzzling finding may be taken as implying that TBP and TFIIB are in different arrangements in PICs formed on mRNA and snRNA promoters. We think it more likely that the arrangement is in fact very similar but that in Pol II snRNA promoters, it is reached through sequential binding of TBP and TFIIB to a SNAPc–PSE complex with rearrangements of protein–protein interactions in the process. Second, addition of TBP to the binding reaction renders the recruitment of BRF2 TATA-box-sensitive: BRF2 recruitment is prevented in the absence of a TATA box (Fig. 7, arrows labeled c) and enhanced in its presence (Fig. 7, arrows labeled d). Thus, the addition of TBP to an in vitro protein–protein interaction system is sufficient to not only ensure proper BRF2 recruitment on TATA-containing probes but also prevent improper BRF2 recruitment on TATA-less probes.

Why is a BRF2–TBP complex unable to bind to SNAPc in the absence of a TATA box? The crystal structure of a BRF2–TBP–TATA box complex reveals that, like TFIIB, BRF2 interacts with TBP through the two cyclin-like repeats constituting the core domain (Nikolov et al. 1995; Gouge et al. 2015). In addition, however, BRF2 makes a contact on the opposite side of TBP through a region located in its unique C-terminal extension (Gouge et al. 2015), and the BRF2 region linking the core domain to the C-terminal extension forms a SNAPc interaction domain known as the arch (Gouge et al. 2015). It seems likely that the unique ability of a TATA-bound BRF2–TBP complex to interact with SNAPc results from differences between the TATA-bound and the “off DNA” BRF2–TBP complexes. In particular, the SNAPc-interacting arch of BRF2 might be properly positioned only in a TATA-bound complex; for example, as a result of BRF2–DNA interactions or rearrangement of the C-terminal extension. It also seems likely that the arch, present in BRF2 but not in TFIIB, explains the ability of a BRF2–TBP complex, but not a TFIIB–TBP complex, to bind to a TATA box downstream from the PSE–SNAPc complex in a stringent bead-binding assay in which complexes must be stable enough to withstand extensive washing steps.

### Role of TFIIA in specific Pol II and III PCI assembly

The second key factor in determining specific Pol II or Pol III PIC assembly that we identified is TFIIA, which has been reported previously to be required for Pol II, but not for Pol III, snRNA gene transcription (Kuhlman et al. 1999). We show here that it is indeed enriched at Pol II, but not Pol III, snRNA genes. TFIIA can associate directly with TBP (Supplemental Fig. 3C; Kuhlman et al. 1999) and SNAPc (Fig. 5E). It is probably not an initial determinant of PIC specificity, as a TBP–TFIIA complex can assemble on either a TATA box downstream from a SNAPc–PSE complex (data not shown) or, as shown above (Fig. 5F), on just the SNAPc–PSE complex (Fig. 7, arrows labeled e). Nevertheless, in addition to helping TBP recruitment to TATA-less snRNA genes promoters, it competes with BRF2 for binding to TBP (Fig. 5D), consistent with its interaction surface in TBP overlapping with that required for interaction with BRF2 (Gouge et al. 2015). Thus, TFIIA helps recruitment of TBP to a SNAPc–PSE complex and, at these promoters, prevents erroneous recruitment of BRF2.

Our results further emphasize the concept that, as described recently in yeast (Abascal-Palacios et al. 2018), Pol II and Pol III PICs are remarkably related both architecturally and functionally. Exploiting this resemblance, Pol II and Pol III SNAPc-dependent promoters use a common set of promoter elements (DSE and PSE) and transcription factors (DSE-binding factors, SNAPc, and TBP) to recruit Pol II or Pol III. This study shows at the genomic level that SNAPc-dependent promoters are nevertheless exquisitely specific in their polymerase recruitment and sheds light on the molecular details that allow cells to ensure such polymerase recruitment specificity.

## Materials and methods

### Oligonucleotides

Primers used for cloning and mutagenesis are listed in Supplemental Table 1.

The biotinylated fragment of the *U6* promoter (165 base pairs [bp] long) and its derivatives were obtained by PCR with biotinylated primer.

### Cell culture and transfections

Flp-In T-REx 293 cells were cultured in DMEM containing 10% tetracycline-free fetal calf serum (Bioconcept) and penicillin/streptomycin. Cells were transfected with JetPeI (Polyplus) and selected with 150 mg/mL hygromycin.

### Antibodies and immunoblots

The antibodies used were as follows: α-Flag (Sigma), α-SNAP43 (cs46) (James Faresse et al. 2012), α-SNAP50 (cs302), and α-TBP (SL27). Immunoblots were performed as described (Emran et al. 2006). For Western blots, the primary antibodies were diluted 1:3000.

### Recombinant protein expression and purification

Full-length human TBP, BRF2, TFIIB, and BDP1(1–470) double-tagged with Flag and His tags were cloned in a pSB vector and expressed in the BL21 (DE3) strain of *Escherichia coli* for 4 h at 27°C with 0.4 mM IPTG (isopropyl β-D-1-thiogalactopyranoside). Cells were grown at 37°C in M9ZB medium until the optical density reached 0.6 (A600). The temperature was then reduced, and IPTG was added. Cells were harvested by centrifugation at 3000*g* for 20 min at 4°C, and the pellets were stored at −80°C. Cells were lysed in buffer A1 (10 mM Tris-HCl at pH 8.0, 250 mM NaCl, 1% Triton X-100, 50 mM NaH_2_PO_4_, 20 mM β-mercaptoethanol, 1 mM PMSF, 20 mM imidazole) through sonication and clarified by centrifugation at 14,000*g* for 30 min at 4°C. The extracts were incubated with Ni-NTA Superflow resin for 3 h at 4°C with constant stirring. The beads were washed three times with five bead volumes of buffer W1 (20 mM Tris-HCl at pH 8, 500 mM NaCl, 20% glycerol, 0.1% Tween 20, 20 mM imidazole, 0.5 mM PMSF, 1× complete proteases inhibitor [Roche]), and the bound proteins were eluted with buffer containing 300 mM imidazole. Proteins were dialyzed against buffer B1 (20 mM HEPES at pH 7.9, 100 mM KCl, 5 mM MgCl_2_, 10% glycerol, 0.2 mM EDTA, 1 mM DTT, 0.1% Tween-20).

GST-tagged full-length human TBP was cloned in pET11, and GST-TFIIB was cloned in pGEX4T1. Proteins were expressed in BL21(DE3) *E. coli* for 4 h at 27°C, and cells were harvested, resuspended in buffer A2 (25 mM HEPES at pH 7.9, 100 mM KCl, 20% glycerol, 10 mM β-mercaptoethnol, 0.5 mM PMSF, 1× complete proteases inhibitor [Roche]), and lysed through sonication. Clarified bacterial extract was incubated with glutathione beads (Sigma) for 3 h at 4°C. GST-tagged proteins were either used for GST pull-down assays or subjected to thrombin cleavage and used for in vitro transcription, EMSA, or binding assays.

TFIIA was cloned (see Supplemental Table 1 for primer sequences) and expressed as three independent subunits. TFIIAα had a N-terminal Flag tag and a C-terminal His tag, whereas TFIIAβ and TFIIAγ carried only a C-terminal His tag. The three TFIIA subunits were expressed in *E. coli* for 4 h at 27°C, and the cells were induced with 0.4 mM IPTG. Bacterial pellets were dissolved in buffer A3 (10 mM Tris at pH 8.0, 100 mM NaH_2_PO_4_, 8 M urea). The TFIIA subunits were purified over Ni-NTA columns, quantified, and mixed in equimolar amounts. Native TFIIA was reconstituted by stepwise dialysis (2 h in 8 M urea, 2 h in 2 M urea, 4.5 h in 0.5 M urea, and overnight in buffer B1).

SNAPc and SNAPcmini (SNAP190 residues 1–516 and lacking SNAP45) were cloned in pACEBAC1 with a N-terminal Strep tag on SNAP190 and a N-terminal His tag on SNAP50 and expressed in Hi5 cells. Briefly, cells were harvested by centrifugation at 250*g* for 10 min at 4°C. The pellet was resuspended in a buffer containing 750 mM NaCl, 10 mM imidazole, 50 mM HEPES (pH 7.9), 10% glycerol, and 5 mM β-mercaptoethanol (buffer A0). Cells were lysed through sonication, and the supernatant was clarified by centrifugation at 14,000*g* for 1 h at 4°C. The supernatant was loaded on a HisTrap HP column (GE healthcare). The column was then washed with buffer A1 (buffer A0 supplemented with 50 mM imidazole). The proteins were eluted with buffer A0 supplemented with 300 mM imidazole. The elution was then diluted with 1 vol of a buffer containing 50 mM HEPES (pH 7.9), 10% glycerol, and 1 mM DTT before application on a heparin HP column (GE Healthcare). The complex was then eluted with a salt gradient from 250 mM to 1.25 M NaCl. Fractions of interest were pooled, and the His and Strep tags were cleaved overnight at 4°C by treatment with TEV protease and dephosphorylated with λ phosphatase. Imidazole (30 mM) was added to the protein before applying to a HisTrap HP column (GE Healthcare). The flowthrough was concentrated before application on a Superdex 200 16/600 equilibrated with a buffer containing 100 mM NaCl, 50 mM HEPES (pH 7.9), 10% glycerol, and 1 mM TCEP.

### PIC assembly on a U6 promoter

Biotinylated DNA fragments corresponding to the *U6* promoter or promoters with mutated PSE, mutated TATA box, or both elements mutated were incubated with various proteins as indicated in the figures in buffer B1 (20 mM HEPES at pH 7.9, 100 mM KCl, 5 mM MgCl_2_, 0.1% Tween-20, 10% glycerol, 0.2 mM EDTA, 10 mM DTT, 0.1 mg/mL BSA, 40 ng/mL competitor DNA, 1 ng/mL poly dGdC, 1× complete protease [Roche]) for 30 min at room temperature, the protein–DNA complexes were then captured on Dynabeads M-280 (Invitrogen) for 30 min at 4°C, and the bead-bound complexes were washed extensively. The proteins attached to beads were then analyzed by Western blot.

### DNase I footprinting

A probe 5′ end-labeled with [γ-^32^P] ATP containing the mouse *U6* PSE was used. The binding reactions were incubated for 20 min at room temperature and contained radiolabeled DNA probe, the proteins indicated in Figure 2C, and 70 mM KCl, 20 mM HEPES (pH 7.9), 5 mM MgCl_2_, 0.1 mM EDTA, 20 µg of calf serum, 0.8 µg each of pUC118 and poly (dI–dC), 2% polyvinyl alcohol, 10% glycerol, and 1.5 mM DTT. The DNase I digestion was carried out as described (Schmidt et al. 1989). The reaction products were analyzed on a 7% polyacrylamide–urea gel.

### EMSAs

The EMSAs were performed with the components described in the figure legends as described in Gouge et al. (2017) for Figure 5F and as described in Saxena et al. (2005) for Figures 2 and 6. The total binding reaction volumes were 20 µL, and the samples were incubated for 30 min at 30°C.

### In vitro transcription assay

In vitro transcription assays were performed as described (Hu et al. 2003) with minor modifications. Tagged Pol III complex, recombinant SNAPc, recombinant TBP, BRF2 or its derivatives, and recombinant BDP1 were mixed with 250 ng of poly[(dG–dC)–(dG–dC)] and 250 ng of *pU6/Hae/RA.2* construct in 2% glycerol, 5 mM HEPES (pH 7.9), 200 mM ammonium acetate, 5 mM MgCl_2_, 0.025 mM EDTA, 1 mM DTT, and protease inhibitors tablets (Roche).

### ChIPs

ChIPs were performed as described in Orioli et al. (2016). Chromatin was sheared with a Bioruptor sonicator (Diagenode). The ChIPs were performed with anti-Flag antibody (Sigma) and sonicated chromatin from 15 million cells. The sequences of qPCR primers used after ChIP are listed in Supplemental Table 2. Means ± SD of immunoprecipitation/input enrichment of three independent experiments were plotted. Hypothesis testing was performed with two-tailed *t*-test.

### Tag density accumulation profiles

Genes encoding small noncoding RNAs (as obtained from filtering the entries in the University of California at Santa Cruz RNA annotation table with a “snRNA” tag) were split into two groups depending on the presence or absence of a TATA box 25–35 bp upstream of the TSS. The search for the TATA-box motif was performed with the FindM tool (http://ccg.vital-it.ch/ssa/findm.php) with a *P*-value threshold set at 0.001. The genes in each group were further filtered by TBP occupancy at the TSS to select the active ones, a remapping of the TBP Chip-exo (ChIP using λ exonuclease to digest transcription factor-unbound DNA after ChIP) data from Pugh and Venters (2016) to the Hg19 genome version, and calculation of the average genome coverage per 400-bp bin. tRNA genes were categorized into active and silent genes based on previously published data (Orioli et al. 2016), and only active tRNA genes were used for tag density analysis. The resulting lists of genes used in further analysis are shown in Supplemental Table 3. The *RPPH1* gene was analyzed separately.

Chip-seq data were downloaded from Encode or the Gene Expression Omnibus repository as indicated in Supplemental Table 4. Reads were aligned on the Hg19 genome version with STAR (Dobin et al. 2013), and uniquely mapped reads were processed with the Homer software (Das et al. 2010) to calculate tag accumulation density.

### Protein structures visualization

Protein structures were visualized with Pymol 1.8.2.

### Pull-down assay

Protein (0.5–3 µg) was immobilized on either glutathion (Sigma), Strep-Tactin (Qiagen), or Flag (Sigma) beads; incubated with protein analyte in buffer B2 (20 mM HEPES at pH 7.9, 150 mM NaCl, 5 mM MgCl_2_, 0.1% Tween-20, 10% glycerol, 0.2 mM EDTA, 10 mM DTT, 0.1 mg/ml BSA, 1× complete protease [Roche]); and incubated for 1 h at 4°C. The resulting protein complexes were washed four times, and their composition was analyzed by Western blot.

## Supplementary Material

Supplemental Material
