# Supplementary material for: Mechanism of selective recruitment of RNA polymerases II and III to snRNA gene promoters
Source: Genes Dev. 2018 May 1;32(9-10):711–22. doi: 10.1101/gad.314245.118 (PMC6004067; doi:10.1101/gad.314245.118)
Supplement: Supplemental Material [file supp_32_9-10_711__index.html]

Mechanism of selective recruitment of RNA polymerases II and III to snRNA gene promoters — Mechanism of selective recruitment of RNA polymerases II and III to snRNA gene promoters — Supplemental Material 

# Mechanism of selective recruitment of RNA polymerases II and III to snRNA gene promoters

## Supplemental Material

- Supplemental\_Table\_S3.xls
- Supplemental\_Table\_S4.xlsx
- Supplemental\_Material.docx
- Supplemental\_Fig\_S1.pdf
- Supplemental\_Fig\_S4.pdf
- Supplemental\_Fig\_S2.pdf
- Supplemental\_Table\_S1.docx
- Supplemental\_Fig\_S3.pdf
- Supplemental\_Table\_S2.docx
