# Supplementary material for: Mechanism of selective recruitment of RNA polymerases II and III to snRNA gene promoters
Source: Genes Dev. 2018 May 1;32(9-10):711–22. doi: 10.1101/gad.314245.118 (PMC6004067; doi:10.1101/gad.314245.118)
Supplement: Supplemental Material [file supp_gad.314245.118_Supplemental_Material.docx]

**SUPPLEMENTAL MATERIAL**

**Supplemental Figure 1.** Related to Figure 1.

**Supplemental Figure 2.** Related to Figure 4

**Supplemental Figure 3.** Related to Figure 5

**Supplemental Figure 4.** Related to Figure 6C.

**Supplemental Table 1**. Related to Materials and Methods, section "oligonucleotides".

**Supplemental Table 2.** Related to Materials and Methods, section "chromatin immunoprecipitations".

**Supplemental Table 3.** Related to Materials and Methods, section "Tag density accumulation profiles".

**Supplemental Table 4.** Related to Materials and Methods, section "Tag density accumulation profiles".
