# Supplementary material for: Mechanism of selective recruitment of RNA polymerases II and III to snRNA gene promoters
Source: Genes Dev. 2018 May 1;32(9-10):711–22. doi: 10.1101/gad.314245.118 (PMC6004067; doi:10.1101/gad.314245.118)
Supplement: Supplemental Material [file supp_gad.314245.118_Supplemental_Fig_S1.pdf]

Supplemental Dergai_Fig.1

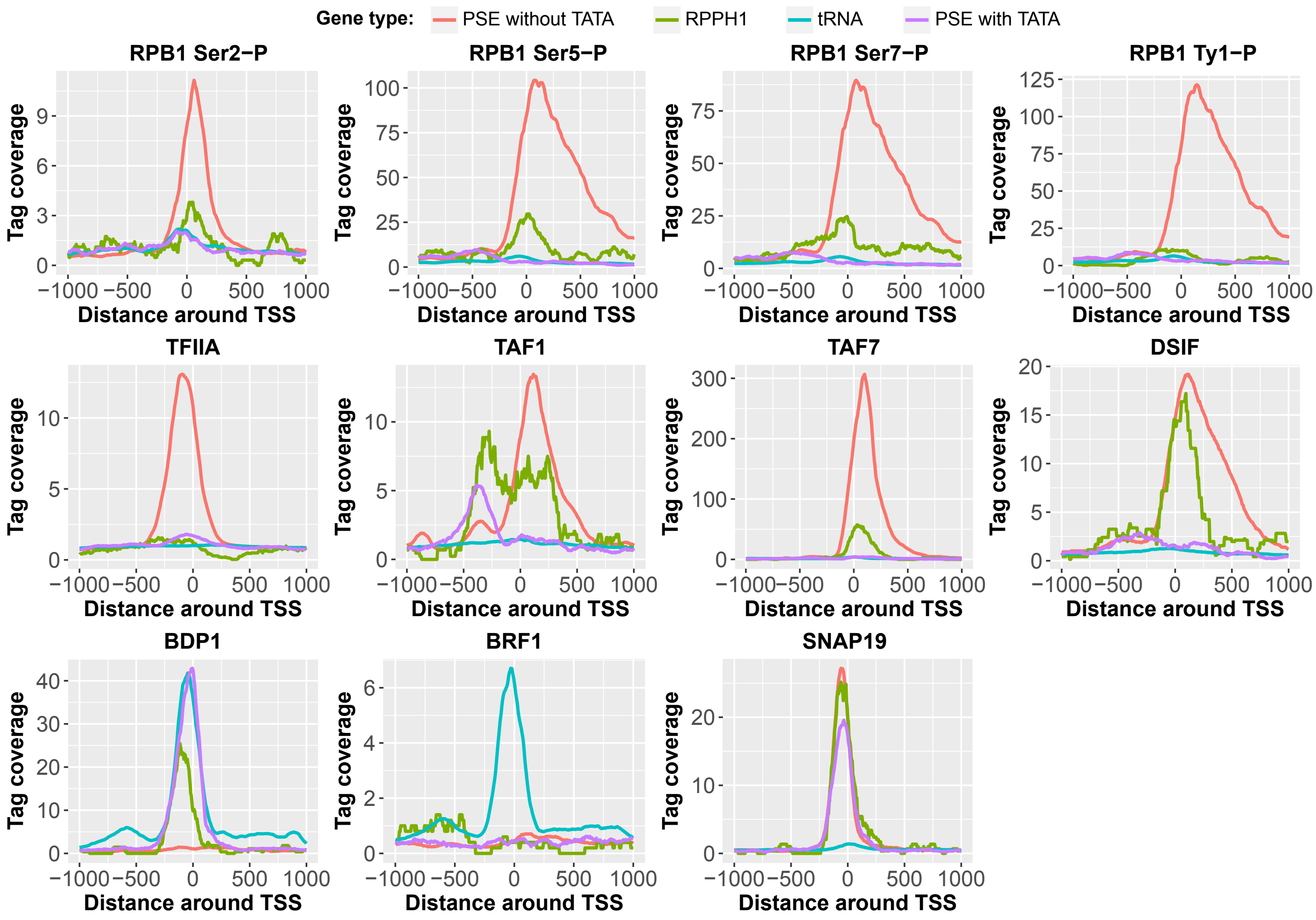

Supplemental Figure 1. Tag density plots for indicated proteins around the TSSs of various gene families. Annotated PSE-containing genes were split into TATA-containing (purple) and TATA-less (red) genes and only active genes, as determined by TBP occupancy, were kept for further analysis. The RPPH1 gene (olive green) was analyzed separately. Only active annotated tRNA genes (turquoise), as determined by Pol III occupancy, were analyzed. See Materials and Methods for further details.
