# Supplementary material for: Mechanism of selective recruitment of RNA polymerases II and III to snRNA gene promoters
Source: Genes Dev. 2018 May 1;32(9-10):711–22. doi: 10.1101/gad.314245.118 (PMC6004067; doi:10.1101/gad.314245.118)
Supplement: Supplemental Material [file supp_gad.314245.118_Supplemental_Fig_S4.pdf]

Supplemental Dergai_Fig.4

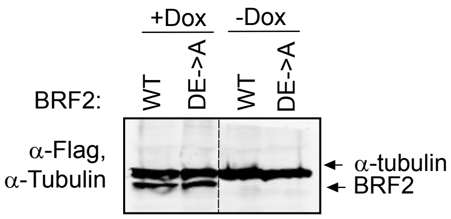

Supplemental Figure 4. A. Immunoblot detection with an anti-Flag antibody of Flag tagged wild-type and mutant BRF2 in extracts from 293 doxycycline-inducible cell lines before and after doxycycline induction as indicated on top of the figure. Alpha-tubulin was detected with an anti-alpha tubulin antibody and the signal used as a loading control.
