# Supplementary material for: Mechanism of selective recruitment of RNA polymerases II and III to snRNA gene promoters
Source: Genes Dev. 2018 May 1;32(9-10):711–22. doi: 10.1101/gad.314245.118 (PMC6004067; doi:10.1101/gad.314245.118)
Supplement: Supplemental Material [file supp_gad.314245.118_Supplemental_Fig_S2.pdf]

Supplemental Dergai_Fig.2

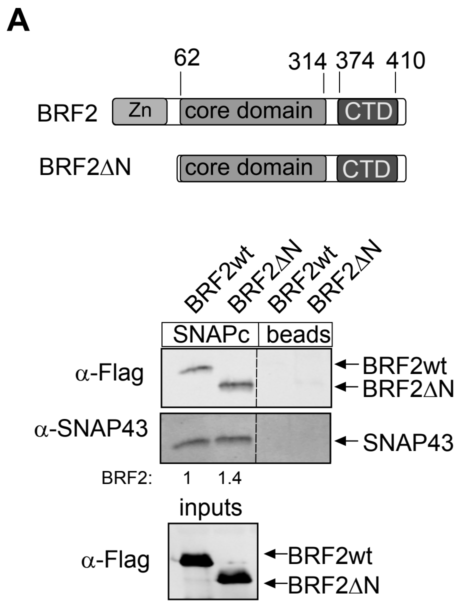

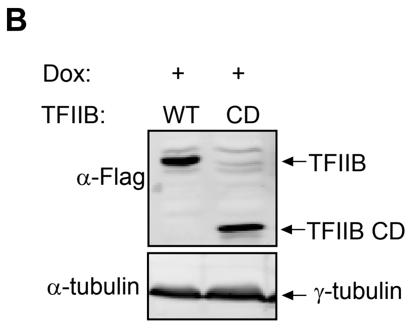

Supplemental Figure 2. A. SNAPc-coated beads (lanes 1, 2) or beads alone (lanes 3, 4) were mixed with the BRF2 proteins indicated on top. Bound proteins were detected by immunoblot with the antibody indicated on the left. The lower panel shows input Flag-tagged BRF2 proteins detected with an anti-Flag antibody. B. Expression of TFIIB and TFIIB core domain in extracts from 293 doxycycline-inducible cell lines after 24 h doxycycline induction.
