# Supplementary material for: Mechanism of selective recruitment of RNA polymerases II and III to snRNA gene promoters
Source: Genes Dev. 2018 May 1;32(9-10):711–22. doi: 10.1101/gad.314245.118 (PMC6004067; doi:10.1101/gad.314245.118)
Supplement: Supplemental Material [file supp_gad.314245.118_Supplemental_Table_S1.docx]

**Supplementary Table 1**

| Primers used for cloning or mutagenesis | |
| --- | --- |
| Oligonucleotide name | Sequence |
| BRF2-A311-Rev | AGGGATCCGGCAGAGCGGACCAGTGA |
| BRF2-D289-Rev | AGGGATCCGTCAAGTCTCAGAACTCG |
| BRF2-386/388A-For | GAACATTTCTGCTAGTGAAATAGCACAGTATTTGCG |
| BRF2-386/388A-Rev | CGCAAATACTGTGCTATTTCACTAGCAGAAATGTTC |
| BRF2-386/388K-For | GAACATTTCTAAAAGTGAAATAAAACAGTATTTGCG |
| BRF2-386/388K-Rev | CGCAAATACTGTTTTATTTCACTTTTAGAAATGTTC |
| BRF2-pVin-For | GCGGTACCATGGACTACAAAGACGATGACGA  CAAGCCAGGCAGAGGCCGC |
| BRF2-pVin-Rev | GCCTCGAGTCAGGGAGGGTTAGGGAC |
| TF2B-pVin-For | GCCTCGAGTCAGGGAGGGTTAGGGAC |
| TF2B-pVin-Rev | ATGGGCCCTTACTTGTCGTCATCGTCTTTGTAGTCTAGCTGTGGTAGTTT |
| TF2B-CD-For | ATCCCGGGAAGATGTACCAGAATCGGAGAACA |
| TF2B-M1-For | GTATCAGCTATTTCTGCTGCAGAAATTGGTCGGTGTTTT |
| TF2B-M1-Rev | AATTTCTGCAGCAGAAATAGCTGATACGGCACATATTTC |
| TF2B-M2-For | ACCGCTGCGGCTGCGGCTACAACTGGGGACTTCATGTCC |
| TF2B-M2-Rev | AGTTGTAGCCGCAGCCGCAGCGGTTTCTAGCGCTTTCAA |
| TF2B-M3-For | GACTTGGCTGCTGCGGCGGCCCCCATCTCTGTGGCAGCG |
| TF2B-M3-Rev | GATGGGGGCCGCCGCAGCAGCCAAGTCCAATTCCACAGC |
| TF2B-R169E-For | CTCTATATTGCCTGTGAACAAGAAGGGGTTCCT |
| TF2B-R169E-Rev | AGGAACCCCTTCTTGTTCACAGGCAATATAGAG |
| TBP-pSB-For | GCTCTAGAACTCCCGGAATCCCT |
| TBP-pSB-Rev | GCGGATCCCGTCGTCTTCCTGAA |
| TBP-M1-For | ATAAGGTTAGCAGGCCTTGTGGCCACCCACCAA |
| TBP-M1-Rev | TTGGTGGGTGGCCACAAGGCCTGCTAACCTTAT |
| TBP-M2-For | AGTGCTGCGGCAGCGGCAGCTCCTGGTTTAATC |
| TBP-M2-Rev | AGCTGCCGCTGCCGCAGCACTACTAAATTGTTG |
| TBPstop286F | CAGCAGCAGCAACAGTGAGTGGCAGCTGCAGCC |
| TBP stop286R | GGCTGCAGCTGCCACTCACTGTTGCTGCTGCTG |
| TBPstop472F | ACGCCAGCTTCGGAGTGATCTGGGATTGTACCG |
| TBPstop472R | CGGTACAATCCCAGATCACTCCGAAGCTGGCGT |
| TBP_CD_For | TCG GAG AGT TCT GGG ATT G |
| TBP_CD_Rev | TCT AGA TCC ACG CGG AAC |
| TF2A α For | GCTCTAGAGCGAACTCGGCAAATACA |
| TF2A α Rev | GCGGATCCCTCTTTGTCTTCCTCCTC |
| TF2A β For | GCTCTAGAAAAGATGGAGCTGAAGAT |
| TF2A β Rev | GCGGATCCCCATTCTGCATCTCCAAT |
| TF2A γ For | GCTCTAGAGCATATCAGTTATACAGA |
| TF2A γ Rev | GCGGATCCTTCTGTAGTATTGGAGC |
