# Supplementary material for: Mechanism of selective recruitment of RNA polymerases II and III to snRNA gene promoters
Source: Genes Dev. 2018 May 1;32(9-10):711–22. doi: 10.1101/gad.314245.118 (PMC6004067; doi:10.1101/gad.314245.118)
Supplement: Supplemental Material [file supp_gad.314245.118_Supplemental_Fig_S3.pdf]

Supplemental Dergai_Fig.3

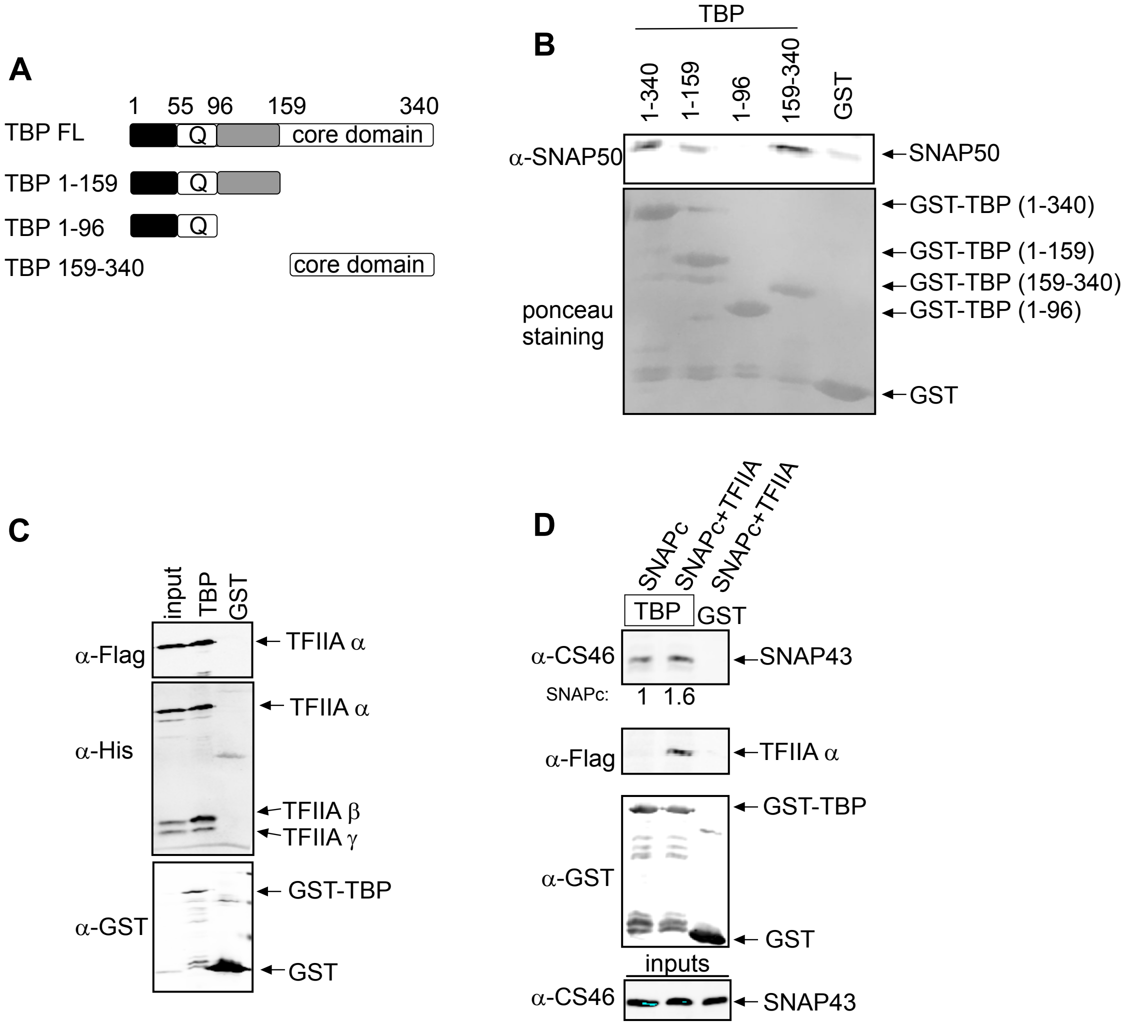

Supplemental Figure 3. A. Structure of TBP truncations. B. Upper panel. SNAPc retained on various GST-TBP truncations or GST alone, as indicated on top of the figure, was detected by immunoblot with an anti-SNAP50 antibody. Lower panel shows bait protein as detected by ponceau staining. C. TFIIA was incubated with GST-TBP (lane 2) or GST (lane 3). Lane 1 shows input TFIIA. Input (lane 1) or bound proteins (lanes 2 and 3) were detected by immunoblot with the antibodies indicated on the left. D. GST-TBP (Lanes 1, 2) or GST (lane 3) were immobilized on beads and incubated with SNAPc alone or complemented with TFIIA. Bound proteins were detected by immunoblot with the antibodies indicated on the left. The bottom panel shows input SNAPc as detected with an anti-SNAP43 antibody.
