# Supplementary material for: Mechanism of selective recruitment of RNA polymerases II and III to snRNA gene promoters
Source: Genes Dev. 2018 May 1;32(9-10):711–22. doi: 10.1101/gad.314245.118 (PMC6004067; doi:10.1101/gad.314245.118)
Supplement: Supplemental Material [file supp_gad.314245.118_Supplemental_Table_S2.docx]

**Supplemental Table 2**

| Primers used for ChIP-qPCR | |
| --- | --- |
| Oligonucleotide name | Sequence |
| **RNU6-F** | **GCACGAAAGTTGTTCTGCAA** |
| **RNU6-R** | **AGGGGCCATGCTAATCTTCT** |
| RNU2-For | AAACACGCGTCATTCAACAC |
| RNU2-Rev | CACGCATCGACCTGGTATT |
| Gene-desert-For | AAACCCTGGCACATAAGCAC |
| Gene-desert-Rev | AAGGGTGGAGCTGAACTTGA |
